# Phosphate availability affects fixed nitrogen transfer from diazotrophs to their epibionts

**DOI:** 10.1038/s41396-019-0453-5

**Published:** 2019-06-27

**Authors:** Niels J. Schoffelen, Wiebke Mohr, Timothy G. Ferdelman, Julia Duerschlag, Sten Littmann, Helle Ploug, Marcel M. M. Kuypers

**Affiliations:** 1 Department of Biogeochemistry, Max Planck Institute for Marine Microbiology, Celsiusstraße 1, 28359 Bremen, Germany; 2 Department of Marine Sciences, University of Gothenburg, Carl Skottsbergs Gata 22B, 41319 Gothenburg, Sweden

**Keywords:** Biogeochemistry, Microbial ecology

## Abstract

Dinitrogen (N_2_) fixation is a major source of external nitrogen (N) to aquatic ecosystems and therefore exerts control over productivity. Studies have shown that N_2_ -fixers release freshly fixed N into the environment, but the causes for this N release are largely unclear. Here, we show that the availability of phosphate can directly affect the transfer of freshly fixed N to epibionts in filamentous, diazotrophic cyanobacteria. Stable-isotope incubations coupled to single-cell analyses showed that <1% and ~15% of freshly fixed N was transferred to epibionts of *Aphanizomenon* and *Nodularia*, respectively, at phosphate scarcity during a summer bloom in the Baltic Sea. When phosphate was added, the transfer of freshly fixed N to epibionts dropped to about half for *Nodularia*, whereas the release from *Aphanizomenon* increased slightly. At the same time, the growth rate of *Nodularia* roughly doubled, indicating that less freshly fixed N was released and was used for biomass production instead. Phosphate scarcity and the resulting release of freshly fixed N could explain the heavy colonization of *Nodularia* filaments by microorganisms during summer blooms. As such, the availability of phosphate may directly affect the partitioning of fixed N_2_ in colonies of diazotrophic cyanobacteria and may impact the interactions with their microbiome.

## Introduction

The availability of dissolved inorganic nitrogen (DIN) often limits primary productivity in aquatic ecosystems. The activity of dinitrogen (N_2_)-fixing microorganisms (diazotrophs) alleviates the nitrogen (N) limitation either directly when diazotrophs are also photoautotrophs or indirectly through the release of fixed N to the microbial community. This fixed N can be released or transferred to the microbial community via several pathways, ranging from cell death [1], viral lysis [2, 3], break-up of cells during grazing [4], to active or passive release of inorganic and organic nitrogen compounds (e.g., ref. [5]). The nitrogenous compounds are then available to other microorganisms [6–10] and can subsequently fuel the activity and growth of higher trophic levels [9, 11–15]. In addition, diazotrophically fixed N can also be exported from the upper water column either by grazing and subsequent transport of fecal pellets to deeper waters, direct export through sinking (e.g., diatom–diazotroph associations [16]), or aggregation and export of marine snow aggregates. Whether diazotrophically fixed N is channeled through the microbial loop or is exported directly could then have profound effects on the turnover and productivity of surface waters [9, 14, 17, 18].

A large portion of N_2_ fixation in aquatic ecosystems is carried out by filamentous cyanobacteria that can form millimeter-large, visible colonies. The non-heterocystous, colonial *Trichodesmium* sp. has been suggested to account for up to half of the marine N_2_ fixation [19–21], while the heterocystous *Aphanizomenon* sp. and *Nodularia* sp. are important contributors to N_2_ fixation in brackish and freshwater environments (e.g., refs [22–24]). Although grazing of filamentous diazotrophs and direct export of their biomass to deeper waters has been observed (e.g., refs [12, 25]), the majority of its fixed N appears to be channeled through the microbial loop and thereby fuels productivity in surface waters [12, 26, 27] with significant contributions to net production in some environments [16, 28].

A relatively large fraction of the total N_2_ fixed might be released to the surrounding water or microbial community. Several field and culture studies have reported the production of dissolved N compounds (often as ammonium or amino acids) from N_2_ fixation and/or their transfer to other organisms (e.g., [5, 6, 9, 29]). The observed release is often higher in natural environments [30] than in culture studies [31, 32], presumably due to the absence of factors potentially promoting N release in culture experiments (such as grazing, nutrient limitation, stress [32]). A co-culture study observed that the growth of both microbial partners (an N_2_-fixer and a non-N_2_-fixer) was promoted through N release relative to the growth in mono-cultures [33], suggesting that N release by an N_2_-fixer could be mutually beneficial in a natural microbial community. The causes for N release in natural environments are, however, often not known. Studies on photosynthesis have put forward several mechanisms for the causes on the release of dissolved organic carbon (DOC), including passive diffusion, photosynthetic overflow, autocatalytic cell death, photorespiration, resource acquisition, defense mechanisms, infochemicals, and density reduction [34]. While these may not be directly transferable to N release, these mechanisms might offer some insight into the causes for N release, particularly in photoautotrophic N_2_-fixing microorganisms. Increased release of dissolved organic matter (DOM) from phytoplankton, primarily as photosynthetic overflow [34], previously has been linked to nutrient limitation [35]. Iron (Fe) and phosphorus (P) are well known to impact the activity of diazotrophs [36–38]. Nevertheless, very little is known about the function of nutrient limitation on the release of diazotrophically fixed N_2_. Indirect evidence that the availability of inorganic P (i.e., phosphate or DIP) could affect the release of fixed N has come from increases in DIN concentration during DIP limitation [39]. In view of the sparsity of studies and the difficulty of teasing apart individual organisms and their physiology in environmental studies, the extent, magnitude, and mechanisms of fixed N release in the environment remains mostly unresolved.

The Baltic Sea endures recurring cyanobacterial summer blooms with high N_2_ fixation rates; in fact, it harbors some of the highest rates measured in any aquatic system [24]. In the central Baltic Sea, the main N_2_-fixing organisms are the cyanobacteria *Aphanizomenon* sp., *Dolichospermum* spp., and/or *Nodularia spumigena* (e.g., [23]). *Aphanizomenon* colonies usually contain few epibionts and little is known about their identity [40, 41]. In contrast, *Nodularia* colonies harbor associated microorganisms distinct from the surrounding seawater [42], including members of the proteobacteria and the *Cytophaga*–*Flavobacterium*–*Bacteroides* group [42, 43]. *Nodularia* shows little overgrowth earlier in the season, but dense epibiont colonization at later stages [44, 45], which is accompanied by changes in the structure of the associated microbial community [42]. Closely associated microorganisms isolated from *Nodularia* colonies have been shown to be both beneficial and detrimental to the cyanobacterial growth [43]. The release and transfer of freshly fixed N in these blooms has previously been documented for both cyanobacteria (e.g., ref. [9]), but the underlying mechanisms are unclear.

Considering the very low DIP concentrations encountered in the late summer bloom, the Baltic Sea provided the ideal site to employ a combination of stable isotope incubations and single-cell techniques to (1) determine the degree of P limitation within colonies of the heterocystous diazotrophic cyanobacteria *Aphanizomenon* and *Nodularia*, (2) measure the transfer of fixed N and carbon (C) to associated epibionts of the cyanobacteria, and (3) test whether DIP availability plays a role in the transfer, under low DIP concentrations in a Baltic Sea summer bloom.

## Material and methods

### Sampling and experimental setup

The sampling and experimental setup corresponds to that described in Schoffelen et al. [46]. For context and reference, this section describes the same experiments (with some additional subsampling), which are also summarized as a sketch in Supplementary Fig. S1. Subsamples exclusively presented in Schoffelen et al. [46], for example, colony-based DIP uptake, are not included here. Field sampling was carried out on three occasions in August 2015 (6th, 8th, and 12th August) at the Swedish National Marine Monitoring site B1 (58°48′18″N, 17°37′52″E), located close to the Askö Marine Research Station (Baltic Sea Center) at the south-eastern coast of Sweden, using a motor-driven boat. Surface waters were collected in a 30-L high-density polyethylene container (Nalgene) by submerging the container in the surface water from the side of the boat. Colonies of filamentous cyanobacteria were gathered by lowering a plankton net (mesh size 20 μm) down to ~3 m depth, hauling it up to the surface and transferring the collected material to a 2.5-L polycarbonate bottle until further processing in the laboratory. Both surface waters and collected colonies were transported back to the Askö Marine Research Station within 30 min after sampling. In the first experiment (6th August 2015), the surface water was subsampled for nutrient concentrations and bulk DIP uptake rates using the radiotracer ^33^P (see section below). The second experiment (8th August 2015) was carried out as described for the first experiment with the addition of:

- bulk rates of CO_2_ and N_2_ fixation (see section below),
- incubations for single-cell rates of CO_2_ and N_2_ fixation by cyanobacteria and the transfer of freshly fixed C and N to their epibionts using the stable isotopes NaH^13^CO_3_ and ^15^N_2_, respectively, at in situ DIP concentrations (see section below; here, also trace amounts of ^33^P-DIP were added and the cyanobacterial single-cell DIP uptake rates have been published in [46]), and
- subsamples for cyanobacteria and epibiont characterization (see section below).

The third experiment (12th August 2015) was carried out as described for the first experiment with the addition of single-cell rates of CO_2_ and N_2_ fixation by cyanobacteria and the transfer of freshly fixed C and N to their epibionts using the stable isotopes NaH^13^CO_3_ and ^15^N_2_, respectively, but under artificially increased (+1 μmol L^−1^) DIP concentration. All incubations were carried out in a temperature-controlled and light-controlled room that was set for in situ conditions measured on the sampling day at station B1 at a 1–2 m water depth (17.4–18.0 °C, ~280 μmol photons m^−2^ s^−1^ light irradiance, 18 h:6 h light–dark cycle). All sampling and incubation equipment was cleaned with a phosphorus-free detergent (Decon90, Decon Laboratories Limited) and acid washed (HCl) prior to use to minimize nutrient contaminations during sampling or labeling experiments. The description of nutrient sample collection, analysis, and the nutrient data (DIP) have been published in Schoffelen et al. [46].

### Bulk CO_2_ and N_2_ fixation rates

CO_2_ and N_2_ fixation rates in the bulk seawater were measured following the modified bubble method described by Klawonn et al. [47]. Water collected at station B1 was transferred to four 2.5-L polycarbonate bottles and capped headspace-free. To three of the four bottles, isotopically enriched sodium bicarbonate (NaH^13^CO_3_, ≥98 atom%, Sigma-Aldrich) and N_2_ gas (^15^N_2_, ≥99 atom%, lot number I-17299, Cambridge Isotope Laboratories) were added through the septum caps. The ^15^N_2_ gas was tested for trace contamination of ^15^NH_4_^+^ using the hypobromite oxidation method [48] prior to the experiments, and no contamination was detected. The bottles were gently agitated for 15 min, the gas bubble was then released, and the bottle was again capped headspace-free. Isotopic enrichments of ^13^C (~11.7 atom%) and ^15^N (~4.3 atom%) in the CO_2_ and N_2_ pool were determined using gas chromatography coupled to isotope-ratio mass spectrometry (GC-IRMS; Finnigan Delta Plus) and membrane inlet mass spectrometry (MIMS;In Process Instruments GAM 200), respectively, in subsamples taken at the end of the incubation. The three isotopically enriched bottles and the one untreated bottle (for natural abundance of ^13^C and ^15^N) were all incubated for 24 h at the conditions described above. Incubations were terminated by vacuum filtration of 0.5 L each onto a 25 mm pre-combusted (460 °C, 6 h) GF/F filter (Whatman). The filters were dried (60 °C, 12 h) and stored at room temperature in the dark until further analysis. Elemental and mass spectrometric measurements, analysis, and calculations were done as described in Martínez-Pérez et al. [49].

### Bulk DIP uptake rates

Bulk DIP uptake rates were determined using small polycarbonate bottles filled with 60 mL of bulk water and 150 kBq radiolabeled phosphate (^33^PO_4_^3−^ equivalent to 0.02 nmol/L final concentration, specific activity 111 TBq mmol^−1^, *t*_1/2_ 25.4 days; Hartmann Analytic). Incubations were performed in five replicates with two (background) controls: one with filtered seawater (0.22 µm pore size) and the other heat killed (80 °C, 30 min). Both controls were used to reveal any unspecific binding of the radiotracer to the bottle, filter, or organic material. All incubations were carried out at conditions described above. Subsamples from the radiotracer incubations were taken every 6 h starting at time zero (*t*_0_) to monitor linearity of uptake. The subsamples were filtered through a polycarbonate filter (GTTP, 0.2 µm pore size, 25 mm diameter, Millipore) and washed twice with 5 mL of filtered seawater. The filter was then transferred to a 6 mL scintillation vial containing 5 mL scintillation fluid (Irga-safe plus, PerkinElmer). Total radioactivity was determined from every incubation bottle using 100 µL of water that were directly transferred into a scintillation vial. All samples from one experiment were measured on the same day using a liquid scintillation counter (type: 425-034, Hidex). Bulk DIP uptake rates were calculated using the linear regression for the radiotracer incorporation into biomass between time points (only the linear part was used, see Supplementary Fig. S2), the total amount of radiotracer added, and the DIP concentration in the water. Uptake rates were corrected for radiotracer decay, and unspecific radiotracer binding using the controls.

### Cyanobacteria and epibionts characterization

Cyanobacterial colonies were hand-picked from the plankton net-collected material with the help of a stereomicroscope. Visual characterization was done by epifluorescence microscopy and scanning electron microscopy (SEM). For epifluorescence microscopy of *Aphanizomenon* (one colony), the picked colony was preserved with formaldehyde (1% w/v final concentration) for 30 min at room temperature and rinsed with demineralized water before being transferred onto a polycarbonate filter (GTTP, 0.22 μm pore size, 25 mm diameter, Millipore). The filter was stored frozen at −20 °C until further processing. For epifluorescence microscopy of *Nodularia* (one colony), we used one of the filters from the single-colony incubations (see below). Both colonies were microscopically identified on the filters by their auto-fluorescence signal and the colony-associated microorganisms were visualized by 4′,6-diamidino-2-phenylindole (DAPI) staining using an epifluorescence microscope (Zeiss Axioplan2 and Axio Imager M2).

The SEM analysis was used to determine the number of epibionts per cyanobacterial cell, only considering cells closely associated to the cyanobacteria (also see single-cell section below). The SEM-based epibiont counting was done on the filters obtained from the single-cell incubations (8th August) described below. In random selection, a total of 24 and 8 images containing 32 and 18 cyanobacterial cells and 64 and 251 epibionts were counted for *Aphanizomenon* and *Nodularia* (one colony each), respectively.

The elemental composition of the cyanobacteria was measured using energy-dispersive X-ray spectroscopy (EDS) coupled to the SEM (SEM-EDS). For each *Aphanizomenon* and *Nodularia*, one colony was sampled at the start of the incubation (*t*_0_; only for 8th August), and one of the three colonies incubated for single-cell measurements (*t*_24_) was also used for elemental analysis (see Supplementary Fig. S1; details of analysis and data published in Schoffelen et al. [46]). The penetration depth of this SEM-EDS analysis was ~2 µm, which assures that the obtained signal from the cyanobacteria has minimal interference from the polycarbonate filter [46]. However, the epibionts are much smaller than the cyanobacteria themselves. Therefore, we analyzed the elemental composition (using SEM-EDS) of the epibionts (8 Aug 2018) on colonies that were picked and transferred to silica wafers to avoid interference. These colonies were fixed with formaldehyde (1% w/v final concentration) for 30 min at room temperature, washed with demineralized water, and then transferred to the silica wafers. The wafers were stored frozen at −20 °C until further analysis. From one colony of each *Aphanizomenon* and *Nodularia*, 21 and 33 epibionts across 7 and 7 randomly selected fields of view were analyzed, respectively. No significant differences (*p* > 0.05) in the elemental ratios were detected between epibionts of *Aphanizomenon* and *Nodularia* based on a Mann–Whitney test (for non-normal distributions) and all epibiont data was therefore combined.

### Single-cell C and N uptake rates

Cyanobacteria CO_2_ and N_2_ fixation and epibiont C and N uptake rates were determined using a stable isotope approach followed by nanometer-scale secondary ion mass spectroscopy (nanoSIMS). The batches of incubation seawater were prepared by adding NaH^13^CO_3_ and ^15^N_2_ to a 2.5-L polycarbonate-filled bottle and capped headspace-free as described above but with filtered seawater (0.2 μm pore size). The bottle was agitated for 15 min on a rotary shaker and the gas bubble was released. The isotope-labeled water was carefully transferred to 6-mL Exetainers. Single hand-picked cyanobacterial colonies were then added to the Exetainers, the radiotracer ^33^P was added (see [46]), the Exetainers were closed headspace-free, and were incubated under the conditions described above. Isotopic enrichments of ^13^C and ^15^N in the CO_2_ and N_2_ pool were measured as described above from subsamples of the batches of prepared incubation water (average across experiments of ~11.9 atom% ^13^C, ~1.8 atom% ^15^N were used in the calculation). Per species a single sample (i.e., a single colony) was terminated at *t*_0_ (only 8th August; used for SEM-EDS analysis of cyanobacteria; see [46]) and three samples (i.e., three colonies) after 24 h by fixing the sample with formaldehyde (1% w/v final concentration) for 30 min at room temperature and then filtering onto a gold-sputtered polycarbonate filter (0.22 μm pore size; 25 mm diameter; Millipore). Filters were washed with nanopure water, dried at room temperature, and mounted onto microscope glass slides using a small drop of 0.1% low-melting-point agarose. The microscope slides were stored in the dark at room temperature before single-cell analysis. One of the three colonies sampled after 24 h was later used in the elemental analysis and the other two colonies were reserved for nanoSIMS analysis of the cyanobacteria. NanoSIMS measurements of the epibionts were done on one colony for *Nodularia* and on up to three colonies for *Aphanizomenon*, since here the SEM-EDS filter was also used for nanoSIMS analysis (Supplementary Fig. S1).

#### nanoSIMS analysis

Single-cell C and N uptake activity rates were determined based on the incorporation of DI^13^C and ^15^N_2_ into the cyanobacteria cell biomass and the uptake of released ^13^C and ^15^N compounds by the epibionts using a nanoSIMS 50 L (CAMECA) at the Max Planck Institute for Marine Microbiology in Bremen, Germany. The analysis of the cyanobacterial cells is described in detail in Schoffelen et al. [46]; the following paragraph therefore describes the analysis of the epibionts. *Aphanizomenon* and *Nodularia* filaments were identified based on autofluorescence and morphology and were marked using a laser micro-dissection system (6000B, Leica). Since *Aphanizomenon* filaments have a sparse epibiotic colonization, most of the individual epibionts were later identified by their nanoSIMS signals (e.g., ^12^C^14^N^−^ or ^32^S^−^) while some were additionally imaged prior to nanoSIMS using SEM. However, many microorganisms associated to *Nodularia* were embedded in the colonies’ mucilage (forming multiple layers; Supplementary Fig. S3), which made it difficult to identify them in the SEM and/or nanoSIMS images. We therefore mainly focused on the epibionts in single to double layers around the filaments and epibionts of *Nodularia* were visualized with SEM prior to nanoSIMS analysis so that measurements could be attributed to individual cells.

After loading the sample into the nanoSIMS, marked filaments were pre-sputtered with a Cs^+^ primary ion beam of 300 pA to remove surface contaminations, implement Cs^+^ ions into the samples, and achieve a stable secondary ion emission rate. A primary Cs^+^ ion beam with a current between 1 and 1.5 pA and a beam diameter <100 nm was rastered across the cells for analysis. For individual cells, secondary ion images of ^12^C^−^, ^13^C^−^, ^12^C^14^N^−^, ^12^C^15^N^−^, ^31^P^−^, and ^32^S^−^ were simultaneously recorded using six electron multipliers. The analysis areas were 15 μm × 15 μm in size. An image size of 256 × 256 pixels with a dwell time of 1 ms per pixel was used for the imaging and a minimum of 40 planes were measured per area. To minimize interferences for ^12^C^15^N^−^ the instrument was tuned for high mass resolution (>8000 MRP). All nanoSIMS measurements were analyzed using the Matlab-based software package Look@NanoSims [50] (available at http://nanosims.geo.uu.nl/nanosimswiki/doku.php/nanosims:lans). For every measurement, secondary ion images were drift corrected and accumulated. Regions of interest were drawn around cells using the secondary ion images, and ^13^C/^12^C, ^15^N/^14^N (inferred from ^12^C^15^N^−^/^12^C^14^N^−^), and ^32^S/^12^C ratios were calculated. Detection limits for the ^13^C/^12^C and ^15^N/^14^N ratios were 0.0117 and 0.0043, respectively (for ^13^C/^12^C also see [46]). The nanoSIMS images were correlated with previously recorded SEM images showing the epibionts along the cyanobacterial filaments.

The relative C and N uptake activity of the epibionts was calculated based on the incorporation of ^13^C and ^15^N into the biomass (originating from DI^13^C or ^15^N_2_), natural abundance of ^13^C and ^15^N from the bulk biomass (see bulk CO_2_ and N_2_ fixation section), and the DI^13^C or ^15^N_2_ labeling in the incubation water. Cellular rates of the epibionts were based on a “prolate spheroid” biovolume calculation [51] and a biovolume to cellular C conversion factor [52]. Cellular N content was calculated using the C:N ratio determined from SEM-EDS analysis as described above. Uptake rates might be slightly underestimated due to sample preparation for nanoSIMS analysis [53].

#### C-based and N-based growth rates

Substrate-based growth rates for epibionts and cyanobacteria were determined based on the incorporation of DI^13^C or ^15^N_2_ into the biomass (*X*_B_; corrected for natural abundance) assuming an even distribution of isotopes between cells during cell division as follows:

1 $${\mathrm{Substrate}}-{\mathrm{based}}\;{\mathrm{growth}}\;{\mathrm{rate}}\left( {{\mathrm{day}}^{ - 1}} \right) = \left( {X_{\mathrm{B}}/X_{\mathrm{I}}} \right)\times 2 \;{\times}\; 1/t,$$

with *X*_I_ (corrected for natural abundance) representing the DI^13^C or ^15^N_2_ atom% labeling and *t* the incubation time. A Mann–Whitney test (for non-normally distributed data) was used to determine significant differences in C-based and N-based growth rates between incubation conditions (in situ or DIP amended) including all measured cells.

#### Partitioning of fixed C and N between cyanobacteria and epibionts

In our experiments, we currently cannot mass balance fixed C and N budgets, which would require not only the measurement of fixed C and N lost to the medium but also the monitoring of total biomass of both cyanobacteria and epibionts throughout the incubation. This in itself is difficult given the heterogeneity of the epibiotic coverage of *Nodularia* and methodological complexity. To estimate how the fixed C and N is partitioned between the cyanobacterial cells and their epibionts, we therefore used the median number of epibionts per cyanobacterial cell and the median cellular rates assuming median cell sizes and C:N ratios calculated from all cells of both treatments. Since the relative and cellular rates are based on the isotopic labeling of the substrate, that is, CO_2_ and N_2_, the calculation of transfer of fixed C and N from cyanobacteria to epibionts assumes that the epibionts only receive newly fixed diazotroph-derived C and N. The contribution of epibionts to the total assimilation of C and N among the cyanobacteria and epibionts was calculated as follows:

2 $$	{\mathrm{Epibionts}}\;{\mathrm{C}}\;{\mathrm{assimilation}}\;{\mathrm{of}}\;{\mathrm{the}}\;{\mathrm{total}}\;\left( \% \right) \\ 	\ \ \ =\left({{\,}^{\mathrm{E}}{\mathrm{C}}\;{\times}\;^{\mathrm{E}}n} \right)\!/\!\left( {\left( {{\,}^{\mathrm{E}}{\mathrm{C}}\;{\times}\;^{\mathrm{E}}n} \right) +\; ^{\mathrm{C}}\!{\mathrm{C}}} \right){\times}100,$$

3 $$	{\mathrm{Epibionts}}\;{\mathrm{C}}\;{\mathrm{assimilation}}\;{\mathrm{of}}\;{\mathrm{the}}\;{\mathrm{total}}\;\left( \% \right) \\ 	\ \ \ = \left( {{\,}^{\mathrm{E}}{\mathrm{N}}\;{\times}\;^{\mathrm{E}}n} \right)\!/\!\left( {\left( {{\,}^{\mathrm{E}}{\mathrm{N}}\;{\times}\;^{\mathrm{E}}n} \right) +\; ^{\mathrm{C}}\!{\mathrm{N}}} \right){\times}100,$$

in which ^C^C and ^C^N represent the median C and N assimilation by the cyanobacterial cells, ^E^C and ^E^N represent the median C and N assimilation by the epibionts, which is multiplied by the number of epibionts attached per cyanobacterial cell (^E^*n*). Since it is possible that not only freshly fixed material was released/transferred, we also calculated the transfer assuming that the substrate for the epibionts was a mixture of freshly fixed material and “older biomass,” using the same equations. For this, we assumed that the released C and N from the cyanobacteria, that is, substrate for the epibionts, had an isotopic composition that was equal to the isotopic composition of the cyanobacterial biomass at the end of the incubation.

## Results

### Environmental conditions, bulk CO_2_ and N_2_ fixation, and DIP uptake during the cyanobacterial summer bloom

The environmental conditions of the cyanobacterial bloom were published in Schoffelen et al. [46], but are described here again for better reference and context. Nutrient concentrations monitored throughout 2015 at sampling station B1 showed that DIN was largely depleted by the beginning of May and remained low throughout August (Swedish Meteorological and Hydrological Institute database for monitoring station B1). At the onset of the N_2_-fixing cyanobacterial bloom in August (bulk CO_2_ fixation: 15.5 ± 1.2 µmol C L^−1^ day^−1^; bulk N_2_ fixation: 1.23 ± 0.07 µmol N L^−1^ day^−1^), DIP concentrations dropped from ~150 to only ~30 nmol L^−1^ over the course of 2 days and stayed low for the duration of the bloom. Water conditions were generally calm, surface water temperatures (1–2 m depth) were between 17.4 and 18.0 °C, and sunlight irradiance was high at ~280 μmol photons m^−2^ s^−1^ during the summer bloom. With respect to the filamentous cyanobacterial diazotrophs, *Aphanizomenon* sp. was most abundant followed by *Dolichospermum* sp. and *Nodularia* sp. [46], which is consistent with previous findings [23].

**Fig. 1:**
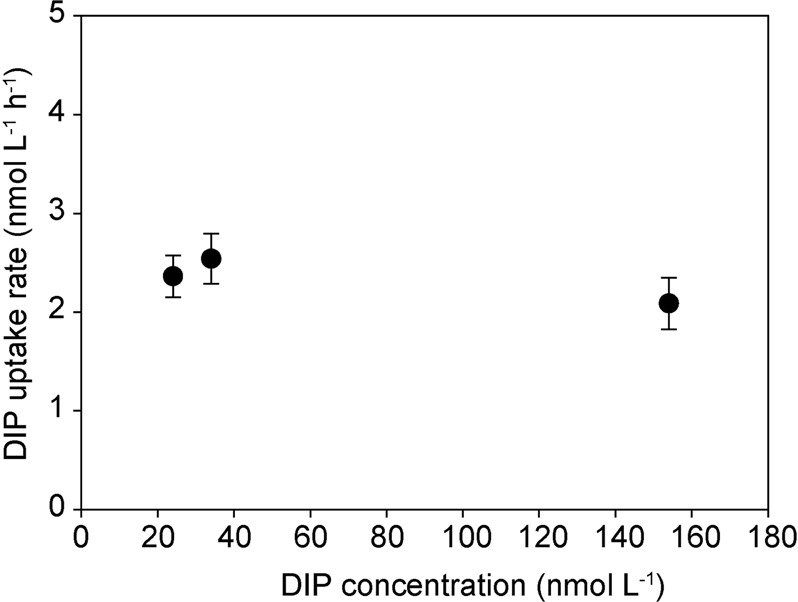
Dissolved inorganic phosphorus (DIP) uptake rates by bulk seawater at different DIP concentrations measured between 6th and 12th August 2015. Symbols and error bars indicate means and standard deviations of five replicate incubations

During the cyanobacterial bloom, DIP uptake rates in the bulk seawater were uniform at 2.1–2.4 nmol L^−1^ h^−1^ during the first 6 h of incubation independent of the initial DIP concentrations of 154, 34, and 23 nmol L^−1^ on 6, 8, and 12th August 2015, respectively (Fig. 1). However, phosphate uptake halted when DIP concentrations dropped below ~10 nmol L^−1^, respectively (Supplementary Fig. S2), indicating that the bulk microbial community starts experiencing DIP limitation below this concentration. The filamentous cyanobacterial colonies, however, already experienced DIP stress at higher DIP concentrations with an apparent *K*_M_ of ~60 nmol L^−1^ [46].

### Epibiont visualization, abundance, and elemental composition

**Fig. 2:**
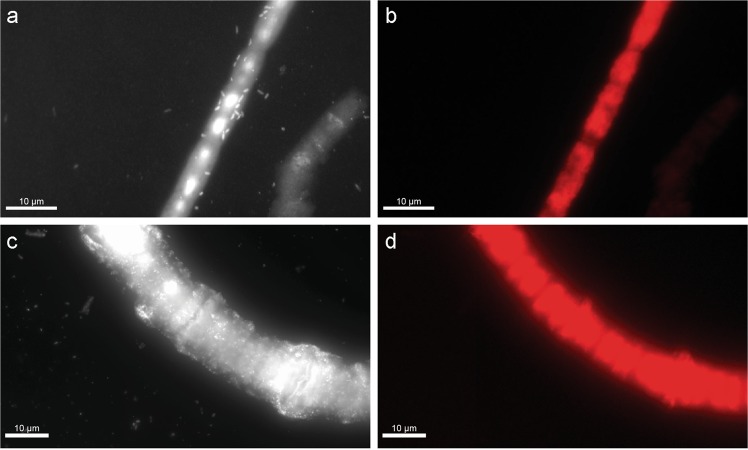
Microscopic images of *Aphanizomenon* (**a**, **b**) and *Nodularia* (**c**, **d**) filaments showing their epibiotic colonization using epifluorescence of 4′,6-diamidino-2-phenylindole (DAPI)-stained cells (**a**, **c**) and autofluorescence (**b**, **d**). Scale bars are 10 μm

**Fig. 3:**
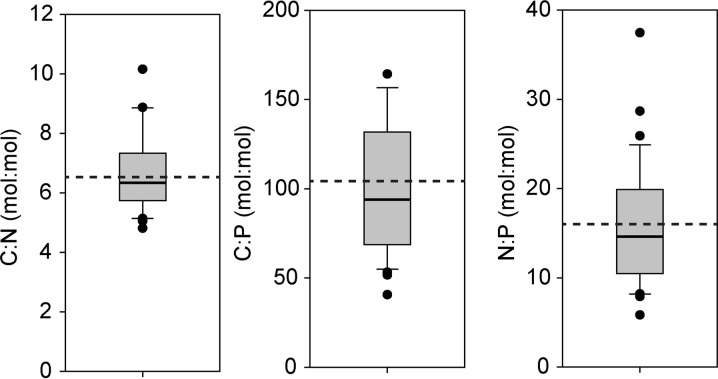
Elemental composition of epibionts (*n* = 54; no significant difference between *Aphanizomenon*-associated (*n* = 21) and *Nodularia* -associated (*n* = 33) epibionts; Mann–Whitney, *p* values C:N = 0.10, C:P = 0.43, N:P = 0.80) as determined by SEM-EDS. Dashed lines indicate the canonical Redfield ratio

Microscopic characterization of picked cyanobacterial colonies (stained with DAPI) revealed mostly non-autofluorescent bacteria associated with filaments of both *Aphanizomenon* and *Nodularia* (Fig. 2, Supplementary Fig. S3). Associated cells occurred primarily as individual cells or occasionally as small clusters of 2–5 cells along *Aphanizomenon* filaments. In contrast, *Nodularia* filaments were densely populated, with large cell clusters often forming a multi-layer biofilm within the mucilage along the filament. Due to this mucilage in the multi-layer biofilm, epibionts were indistinguishable from each other and the organic-rich mucus under SEM and/or nanoSIMS. Therefore, we focused mainly on single-layer or double-layer epibiont biofilms closely associated to the filaments (see Methods section). Associated epibiotic cells had an average size of 0.84 ± 0.11 µm^3^ (median ± SE). The abundance of epibionts was highly variable with median abundances of 2 (mean ± SD of 2.3 ± 1.7; *n* = 24 fields of view) and 20 (mean ± SD of 35.4 ± 35.1; *n* = 8 fields of view) epibionts per cyanobacterial cell for *Aphanizomenon* and *Nodularia*, respectively. Since we only considered closely associated cells on *Nodularia* filaments, our estimate is conservative and the true number of epibionts is likely much higher considering the dense colonization in the mucilage (Fig. 2, Supplementary Fig. S3). Elemental analysis of the epibionts showed C:N, C:P, and N:P ratios slightly below the canonical Redfield ratio (Fig. 3), while the filamentous cyanobacteria had higher elemental ratios (C:P ~130:1 [46]).

### Single-cell C and N uptake by cyanobacterial filaments and their epibionts

**Fig. 4:**
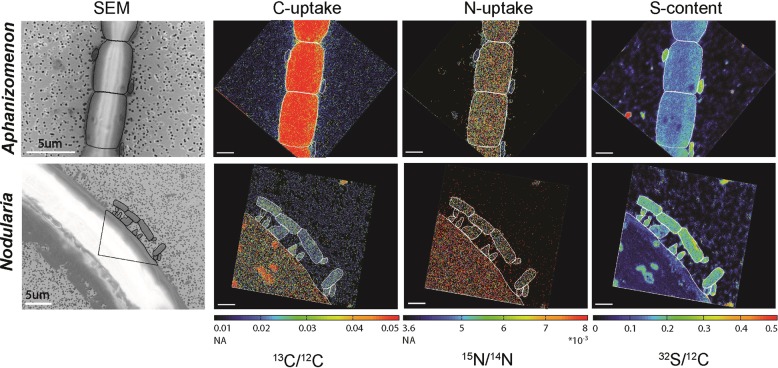
Single-cell imaging of *Aphanizomenon* and *Nodularia* filaments and their epibionts. Single epibionts were identified using either nanometer-scale secondary ion mass spectroscopy (nanoSIMS) (*Aphanizomenon* only) or scanning electron microscopy (SEM; *Nodularia* and *Aphanizomenon*) and their C and N uptake was subsequently measured using nanoSIMS. C and N uptake are shown as ^13^C/^12^C and ^15^/^14^N ratios. Cellular sulfur distribution is shown as the ^32^S/^12^C ratio which also clearly showed the epibionts. Outlines indicate measured cyanobacterial cells and epibionts. NA = natural abundance. NanoSIMS scale bars are 2 μm. NanoSIMS images are superimposed on a black background box to adjust the image orientation relative to the SEM images

**Fig. 5:**
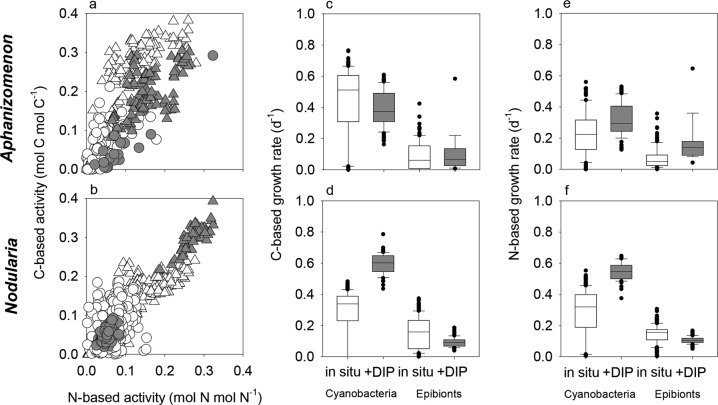
Single-cell C-based and N-based activities and growth rates of *Aphanizomenon* (**a**, **c**, **e**) and *Nodularia* (**b**, **d**, **f**) and their epibionts. **a**, **b** Cyanobacterial (triangles) and epibiotic (circles) relative activities under in situ (white) and added dissolved inorganic phosphorus (DIP) (+1 µM) conditions (gray), respectively. **c**–**f** Growth rates of the cyanobacteria and epibionts for both in situ (white) and added DIP (+1 µM; gray) conditions (lines = medians, boxes = 25th and 75th percentiles, bars = standard deviation, black circles = outliers). In *Aphanizomenon* colonies incubated under in situ conditions, of a total of 153 measured epibionts across 25 fields of view, 111 and 41 cells had ^13^C/^12^C and ^15^N/^14^N ratios, respectively, above detection limit. In the +DIP condition, from a total of 18 measured epibionts across 10 fields of view, 15 and 15 epibionts had ^13^C/^12^C and ^15^N/^14^N ratios, respectively, above detection limit. In *Nodularia* colonies incubated under in situ conditions, of a total of 253 measured epibionts across eight fields of view, 238 and 205 epibionts had ^13^C/^12^C and ^15^N/^14^N ratios, respectively, above detection limit. In the +DIP condition, from a total of 104 measured epibionts across five fields of view, 104 and 86 epibionts had ^13^C/^12^C and ^15^N/^14^N ratios, respectively, above detection limit

Cyanobacterial filaments were highly enriched in ^13^C and ^15^N showing their active ^13^CO_2_ and ^15^N_2_ fixation during the 24-h incubations (Fig. 4; [46]). Epibionts of *Aphanizomenon* showed low enrichments in both isotopes indicating a low transfer of freshly fixed material from *Aphanizomenon* to its epibionts. Relative activities of epibionts were calculated from the enrichments assuming that the transfer of freshly fixed material is the only source of C and N for the epibionts. C-based and N-based relative activities (see Methods section) of the *Aphanizomenon*-associated cells were low at 0.03 ± 0.04 (median ± SD) and 0.03 ± 0.03 (median ± SD) under in situ conditions, respectively (Fig. 5). The transfer of freshly fixed material to the epibionts remained low at 0.03 ± 0.06 and 0.07 ± 0.07 (median  ± SD) for C-based and N-based activity, respectively, when DIP was added to concentrations of ~1 µmol L^−1^ with no significant difference for C (*p* = 0.66) and a significant difference for N (*p* < 0.001), respectively (Fig. 5). Epibionts of *Nodularia* showed higher enrichments in both isotopes, demonstrating a substantial transfer of freshly fixed material (Figs. 4 and 5). Under in situ conditions, the C-based and N-based relative activity of the epibionts was 0.08 ± 0.05 (median ± SD) and 0.08 ± 0.03 (median ± SD), respectively. However, when DIP was added to concentrations ~1 µmol L^−1^, the relative activities of the epibionts dropped significantly (*p* < 0.001 for both C and N) to 0.05 ± 0.02 (median ± SD) and 0.05 ± 0.01 (median ± SD) for C and N, respectively. This drop in the relative activities indicates that less freshly fixed C and N was transferred from *Nodularia* to its epibionts.

To compare the relative activities of the epibionts with the growth rates of the cyanobacteria, the relative activities were translated to C-based and N-based growth rates. The C-based growth rate of *Aphanizomenon* decreased from 0.51 to 0.38 day^−1^ (*p* < 0.001) [46], while the N-based growth rate increased from 0.22 to 0.29 day^−1^ (*p* < 0.001) when DIP was added. The C-based growth rate of its epibionts did not change significantly (*p* = 0.66) while the N-based growth rate increased significantly (*p* < 0.001) (Fig. 5). The response of *Nodularia* and its epibionts, however, was very distinct. *Nodularia* grew significantly faster with added DIP (both C-based and N-based; *p* < 0.001) than under in situ conditions [46]. At the same time, the epibionts’ growth rates declined significantly (both C-based and N-based; *p* < 0.001) (Fig. 5).

## Discussion

Blooms of filamentous diazotrophic cyanobacteria develop annually during summer in the Baltic Sea. While several parameters contribute to the bloom development, they tend to be largely fueled by an excess of DIP in stratified surface waters low in DIN [54]. At the time of our field experiments in 2015, a typical cyanobacterial bloom was developing in early August when DIP concentrations were still around 150 nmol L^−1^. Within a 2-day time window, the DIP concentration decreased to about 20–30 nmol L^−1^ and remained low throughout the bloom. Despite these low DIP concentrations, the majority of the microbial community was still able to obtain sufficient P for growth as indicated by the unchanged DIP uptake rate of the bulk seawater. Our incubations showed that DIP limitation in the bulk community is likely to set in only at concentrations below ~10 nmol L^−1^. This is consistent with estimated *K*_M_ values for bacterial/microbial DIP uptake at concentrations of <20 nmol DIP L^−1^ [55–57]. Previous studies also observed that most of the DIP uptake is carried out by the picoplankton community, which appear to outcompete larger organisms at low DIP concentrations (usually <10 µm size fraction; e.g., [57–64]). Fittingly, the cyanobacterial colonies in the bloom experienced acute DIP stress as DIP concentrations were well below their apparent *K*_M_ [46]. It has to be kept in mind, though, that cyanobacterial colonies can entrap other organisms and have epibiotic communities distinct from the surrounding seawater [42, 43, 65]. While the apparent *K*_M_ value does not distinguish between the different members of the colonies, the elemental ratios of individual cells within a colony can reveal potentially low cellular P content, presumably the result of P stress. The single-cell C:P and N:P ratios of the cyanobacterial filaments showed high elemental ratios (C:P ~130:1 with an increase to ~170:1 within 24 h; [46]) in agreement with the scarcity of DIP. This is in stark contrast to the epibiotic communities. Epibionts of both *Aphanizomenon* and *Nodularia* had elemental ratios slightly below the canonical Redfield ratio (Fig. 3), suggesting that the epibionts were not experiencing P stress at that time.

In the absence of P stress in the epibiotic community, their growth is likely driven by the availability of N and organic C as most of the cells were likely heterotrophic (i.e., absence of autofluorescence). An obvious source of both N and organic C are the cyanobacterial filaments themselves, which have been shown to release inorganic and organic material [5, 6, 9, 29, 40, 44]. In fact, the release of fixed N_2_ as ammonium has previously been shown for both *Aphanizomenon* [40] and *Nodularia* [29]. Although those studies were also carried out in summer, it is unclear if the reported ammonium release was related to the DIP availability since DIP concentrations were not measured.

The epibionts are among the organisms most likely to consume both inorganic/organic C and N that was released due to their proximity to the source that is within the diffusive boundary layer for the epibionts [29, 40]. Any uptake of released C or N by epibionts should therefore relate to the release of freshly fixed material originating from CO_2_ and N_2_ fixation by the cyanobacterial filaments. Although in our measurements we cannot distinguish other potential autotrophic microorganisms from heterotrophs among the epibionts, the microscopic imaging suggests that the vast majority of the epibionts lacked autofluorescence. Our estimate of uptake of fixed C released by the cyanobacteria should therefore be little biased by other autotrophs. Similarly, we cannot exclude that an epibiont may have been an N_2_-fixing microorganism. However, Farnelid and co-authors [66] found that only ~0.7% of *nifH* sequences recovered from size-fractionated biomass >10 µm in surface waters of the Baltic Sea were associated to non-cyanobacterial sequences, indicating that other diazotrophs do not play a substantial role in N_2_ fixation within the colonies. Further, ammonium inside colonies can reach high concentrations [40] that would likely inhibit other diazotrophs in active N_2_ fixation.

Our single-cell analyses using nanoSIMS showed that epibionts of *Aphanizomenon* consumed less released C and N than epibionts of *Nodularia* did, despite both cyanobacteria experiencing DIP stress [46]. We recently found that *Nodularia* and *Aphanizomenon* used different P sources for growth under DIP scarcity during this bloom [46]. Although *Nodularia* is in principle capable of accessing ester-bound organic phosphate and phosphonates via (alkaline) phosphatases and a CP-lyase, respectively [67, 68], they did not use organic P under DIP scarcity in the Baltic Sea during the 2015 summer bloom [46]. In contrast, *Aphanizomenon*, also capable of using organic P via alkaline phosphatases [69], exploited organic P sources to counteract the DIP scarcity [46]. Our observation that *Nodularia* transferred substantially more C and N to epibionts than *Aphanizomenon* could therefore be the direct result of the dependence of growth on DIP and *Aphanizomenon*’s use of organic P. To test this hypothesis, we incubated the field-collected cyanobacterial colonies (including their epibionts) with additional DIP, enough to alleviate the DIP stress. With plenty of DIP available, *Aphanizomenon*’s C-based and N-based growth rates decreased and increased slightly, respectively (Fig. 5; [46]). At the same time, the transfer of C from *Aphanizomenon* to its epibionts remained similar while the transfer of N increased slightly (Fig. 5). However, based on the relatively low activity by the epibionts (Figs. 4 and 5), it is unlikely that *Aphanizomenon* released a large amount of fixed C and/or N under both in situ and increased DIP concentrations. These results support the idea that *Aphanizomenon* used organic P to combat DIP scarcity and thereby prevented the release of larger amounts of fixed C and N. *Nodularia*’s response to increased DIP concentrations was very different, with cells growing significantly faster with added DIP than under DIP scarcity (Fig. 5). Interestingly, at the same time the transfer of fixed C and N to the epibionts significantly decreased with added DIP, indicating that the fixed C and N was used for growth of *Nodularia* rather than being released into the colony. The combined results indicate that the availability of DIP can have a direct effect on the transfer of diazotrophically fixed N_2_ to epibionts, but that this is likely dependent on the prevailing N_2_-fixing species and its specific dependence on DIP for growth.

To assess the potential ecological significance, we calculated the partitioning of fixed CO_2_ and N_2_ between cyanobacteria and epibionts as a function of their growth rate. The resulting transfer rates are presented as a percentage (median ± SE) of the total amount of fixed CO_2_ and N_2_ (i.e., the sum of fixed C and N retained in a cyanobacterial cell and fixed C and N transferred to its epibionts) and should thus not be deemed as exact amounts.

**Fig. 6:**
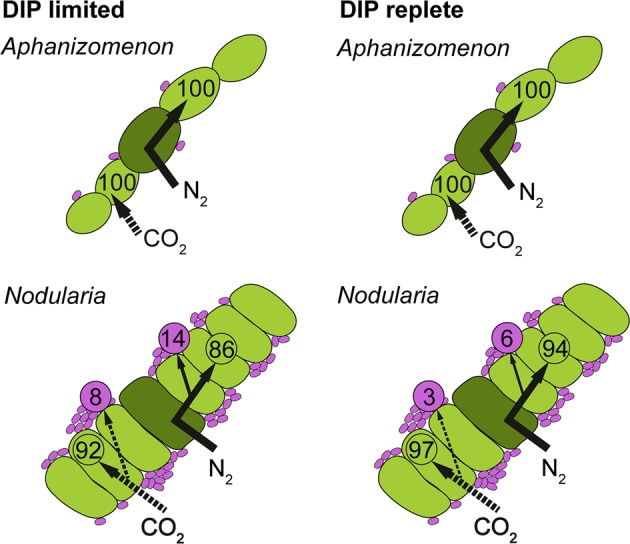
CO_2_ and N_2_ fixation by cyanobacteria and C and N uptake by epibionts as a percentage of the total uptake by the colony (i.e., fixed C and N recovered in the cyanobacteria and their epibionts) under in situ (dissolved inorganic phosphorus (DIP)-limited) and added DIP (+1 µM; DIP-replete) conditions. Arrows indicate pathways of fixation and release of N_2_ (solid arrows) and CO_2_ (dashed arrows). Most of the fixed C and N is retained in the cyanobacterial biomass, but a portion of the fixed material is either actively or passively released and is assimilated by the epibionts. Dark green: heterocysts; light green: vegetative cells; light magenta: epibionts. Only major fluxes are plotted, fluxes below 1% are not included here. Please note here that the partitioning was calculated with cellular rates for cells of median size and C:N ratios across both treatments (see Methods and Results section)

In *Aphanizomenon*, <1% of the fixed C and N was transferred from cyanobacterial cells to their epibionts, suggesting that the transfer of fixed C and N and the overall release was very little (Fig. 6). This small percentage was not only due to the low number of epibionts but also the lower uptake per cell (Supplementary Fig. S4). The total transfer increased only slightly (still ≤1% for C and N) when more DIP was added, suggesting that, at least at the time of our sampling, the use of organic P by *Aphanizomenon* likely prevented the release and transfer of fixed material. In contrast to *Aphanizomenon*, the amount of fixed C and N taken up by epibionts of *Nodularia* was 8 ± 5% and 14 ± 9% of the total amount fixed per cyanobacterial cell, respectively (Fig. 6). However, when DIP was added, this transfer dropped to 3 ± 2% and 6 ± 4% of the total fixed C and N, respectively. These estimates of transfer are conservative, and the total transfer (and release) was likely higher for two reasons. First, we determined only the transfer of freshly fixed C and N. The release and transfer rates would have been higher if a mixture of older material and freshly fixed C and N was released. We calculated the transfer rates of fixed C and N assuming that the released material had an isotopic composition equal to the median enrichment of the cyanobacterial cells at the end of the incubation, that is, the substrate for epibionts had the isotopic composition of the cyanobacterial biomass. Under in situ conditions, the transfer of fixed C and N from *Aphanizomenon* to its epibionts would then still be <1% and 3 ± 1% of total C and N fixed, respectively. For *Nodularia*, however, the transfer would increase to 35 ± 24% and 51 ± 36% for C and N, respectively, under in situ conditions. Second, we only analyzed the closely associated microorganisms in the single or double layers. It is conceivable that not only these epibionts consumed released C and N but also the other colony-associated microorganisms. Considering the extensive microbial population in the mucilage of *Nodularia* (Fig. 2; Supplementary Fig. S3), the transfer might be several fold higher than what was recovered in epibionts directly attached to the filaments. In summary, our data clearly shows that the availability of DIP can directly affect the release and transfer of freshly fixed C and N in filamentous diazotrophic cyanobacteria, likely to an ecologically significant extent.

The pathway of release/transfer in our experiments is yet unknown. Since the colonies were hand-picked for the incubations, side effects of grazing were likely little. The faster growth response by *Nodularia* and the nearly unchanged growth by *Aphanizomenon* to DIP additions suggests that cell death or viral lysis were unlikely to have caused the release under DIP limitation since both would have probably resulted in the release of DIP/organic P alleviating DIP limitation as well. Moreover, both diazotrophs still showed substantial C-based and N-based growth under DIP scarcity. Our results indicate that the use of distinct P sources by *Aphanizomenon* and *Nodularia* were likely responsible for the observed differences in release. This difference fits to the observed denser colonization of *Nodularia*, which is progressively overgrown by bacteria and other organisms during a bloom [42, 44, 45]. The released C and N likely provided an excellent substrate and matrix for bacterial colonization. It is unclear whether the release of fixed C and N was from leakage or active excretion. Leakage could have resulted from, for example, photosynthetic overflow under nutrient limitation as has been postulated for DOC release [34]. The colonization of filaments by epibionts could therefore be relatively one sided, with epibionts taking advantage of the fixed C and N released due to P stress [70]. On the other hand, colony-forming cyanobacteria such as *Aphanizomenon* and *Nodularia* often harbor diverse communities or epibionts, which are distinct from the bacterioplankton in the surrounding seawater [42, 43]. For the oceanic filamentous cyanobacterium *Trichodesmium*, it has been suggested that the epibiotic community could facilitate the uptake of both Fe and P by *Trichodesmium* through the production and activity of siderophores and alkaline phosphatases [71, 72]. The release of fixed C and N by the cyanobacteria could therefore be a mechanism to attract microorganisms with “food” and eventually benefit from or trigger distinct metabolic activities [18]. With more DIP available, these metabolic activities may no longer be required and transfer decreases. How tightly regulated these interactions are is not quite understood, but experiments on quorum sensing suggest that the activity of the cyanobacteria might be more than just the sum of abiotic factors but rather “a complex interplay of biotic and environmental factors” [73]. The overall capacity of the cyanobacteria to scavenge P might therefore not entirely depend on its own metabolic capacity but also on that of its associated microorganisms or microbiome. The release of fixed C and/or N likely creates a unique microbiome that is based on element cycling [74]. In *Nodularia* colonies, the composition of the microbiome is a dynamic feature throughout the seasons [44, 45] that is possibly linked to the degree of P limitation. Organisms inhabiting oligotrophic environments with continuous nutrient limitation, such as *Trichodesmium*, may harbor a more perpetual microbiome that is tailored to its environment [72].

The capacity of DIP availability to directly affect the release of freshly fixed C and N likely has an impact on how both elements are recycled and/or exported. In contrast to C and N that remains in autotrophic biomass, C and N that is released and processed either by associated microorganisms or bacterioplankton are passed on to different/higher trophic levels with the inherent loss of some of the fixed C as CO_2_. The same is true for epibionts that are grazed upon within colonies [45]. Further, the overgrowth of colonies can change the weight and/or density of colonies [44] and could promote the sinking out of surface waters, directly exporting C and N. Whether the phenomenon observed here could also be found in other areas of the marine environments is unclear. If this is indeed the case, then the availability of DIP to diazotrophs could present a mechanism directly affecting the cycling and/or export of diazotrophically fixed N_2_ in the ocean, and therefore the turnover and productivity of oceanic surface waters.

## Supplementary information

Supplementary Figure Legends

Supplementary Figure S1

Supplementary Figure S2

Supplementary Figure S3

Supplementary Figure S4
