# Supplementary material for: Phosphate availability affects fixed nitrogen transfer from diazotrophs to their epibionts
Source: ISME J. 2019 Jun 27;13(11):2701–13. doi: 10.1038/s41396-019-0453-5 (PMC6794295; doi:10.1038/s41396-019-0453-5)
Supplement: Supplementary file 1 — Supplementary Figure Legends [file 41396_2019_453_MOESM1_ESM.docx]

**Supplementary Figure S1:** Schematic diagram of the experimental approach. Surface seawater (A) and cyanobacterial colonies (B) were collected on 6, 8 and 12 August 2015. Surface seawater was used to determine bulk DIP uptake by the microbial community (1) as well as bulk rates of CO_2_ and N_2_ fixation (2). For the single-colony incubations, filtered seawater (FSW) was enriched with ^13^C-DIC and ^15^N_2_ (3 and 4). Colonies were picked with the use of a stereomicroscope (C) for the single colony incubations (3 and 4), the elemental analysis of the epibionts (5), and the colony imaging (6). On 08 August 2015, the single colony incubations were carried out under in situ DIP concentrations (with trace amounts of ^33^P-DIP; single-cell DIP uptake rates by cyanobacteria are published in Schoffelen et al. [46]). On 12 August 2015, the single colony incubations were carried out under increased DIP conditions (+DIP: 1 µM addition). The dates of the respective experiments are indicated in magenta. Number of replicate incubations and or colony allotments are indicated in or next to the bottle symbols or the subsampling. Carbon-based data that has been published in Schoffelen et al. [46] has been indicated with an asterisk.

**Supplementary Figure S2:** Cumulative DIP assimilated by bulk seawater (black circles) and calculated DIP concentrations (gray triangles) during 24-hour incubations on 06 August (a), 08 August (b) and 12 August (c). The DIP concentrations were measured at the start of the incubation (0 h) and were then computed for all other time points based on the assimilated DIP between each of two time points. DIP assimilation rates are means of five replicate incubations with error bars indicating the standard deviation (smaller than symbol size in most cases).

**Supplementary Figure S3:** Epifluorescence images of *Nodularia* filaments (a-i) with DAPI stained cells (left panels) and autofluorescent cells (right panels). Scale bars are indicated in each panel. Autofluorescent cells other than the filamentous cyanobacteria are indicated with white arrows in a, b, f and g (right panels). Panels b and c are zoom-ins of panel a, panels e are zoom-ins of panels d, and panels i are zoom-ins of panels h. In panels a and d, different cyanobacterial filaments can be seen as chains of autofluorescent, pearl-like cells.

**Supplementary Figure S4:** Cellular C- and N-uptake rates by epibionts of *Aphanizomenon* and *Nodularia* under in situ (white symbols) and +DIP (gray symbols) conditions. In *Aphanizomenon* colonies incubated under in situ conditions, of a total of 153 measured epibionts across 25 fields of view, 111 and 41 cells had ^13^C/^12^C and ^15^N/^14^N ratios, respectively, above detection limit. In the +DIP condition, from a total of 18 measured epibionts across 10 fields of view, 15 and 15 epibionts had ^13^C/^12^C and ^15^N/^14^N ratios, respectively, above detection limit. In *Nodularia* colonies incubated under in situ conditions, of a total of 253 measured epibionts across eight fields of view, 238 and 205 epibionts had ^13^C/^12^C and ^15^N/^14^N ratios, respectively, above detection limit. In the +DIP condition, from a total of 104 measured epibionts across five fields of view, 104 and 86 epibionts had ^13^C/^12^C and ^15^N/^14^N ratios, respectively, above detection limit.
