# Supplementary figures and images for: Phosphate availability affects fixed nitrogen transfer from diazotrophs to their epibionts
Source: ISME J. 2019 Jun 27;13(11):2701–13. doi: 10.1038/s41396-019-0453-5 (PMC6794295; doi:10.1038/s41396-019-0453-5)

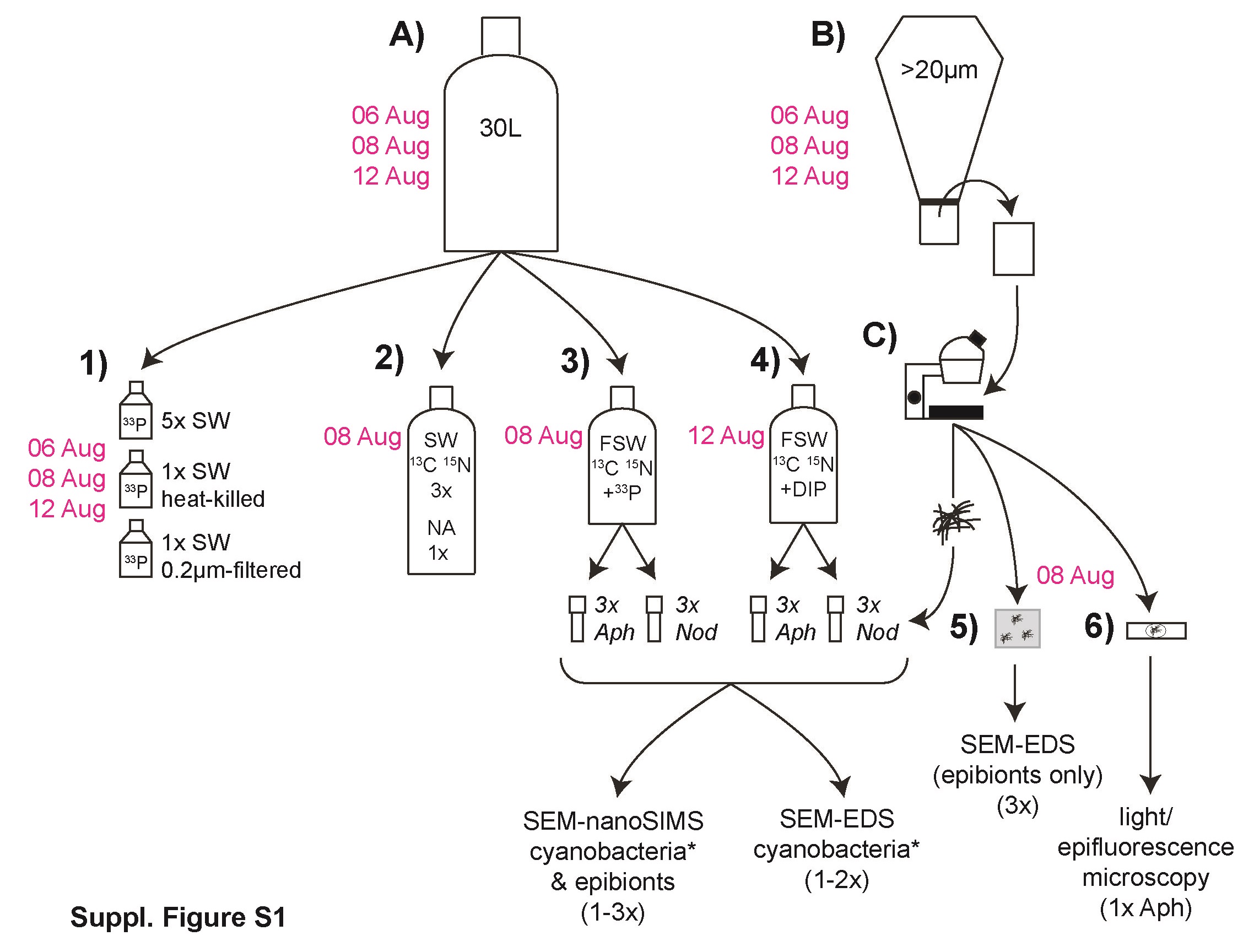

Supplement: Supplementary file 2 — Supplementary Figure S1 [file 41396_2019_453_MOESM2_ESM.jpg]

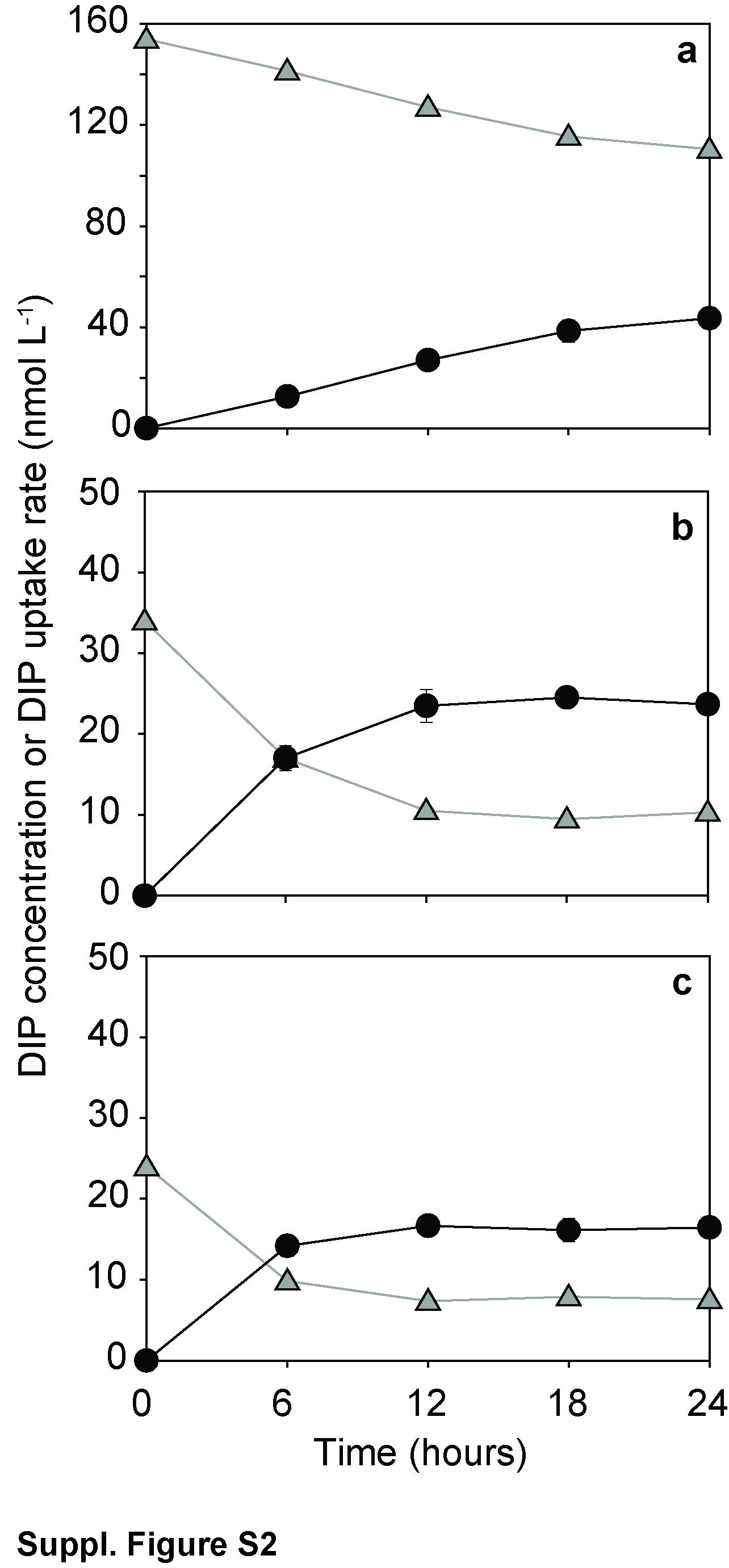

Supplement: Supplementary file 3 — Supplementary Figure S2 [file 41396_2019_453_MOESM3_ESM.jpg]

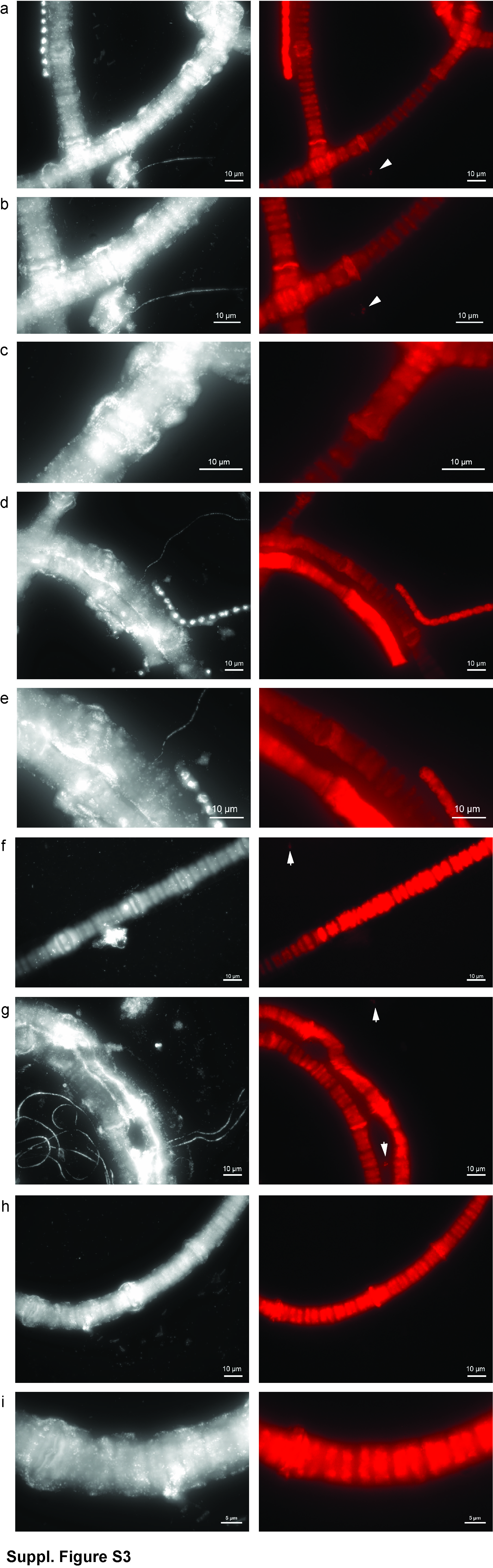

Supplement: Supplementary file 4 — Supplementary Figure S3 [file 41396_2019_453_MOESM4_ESM.jpg]

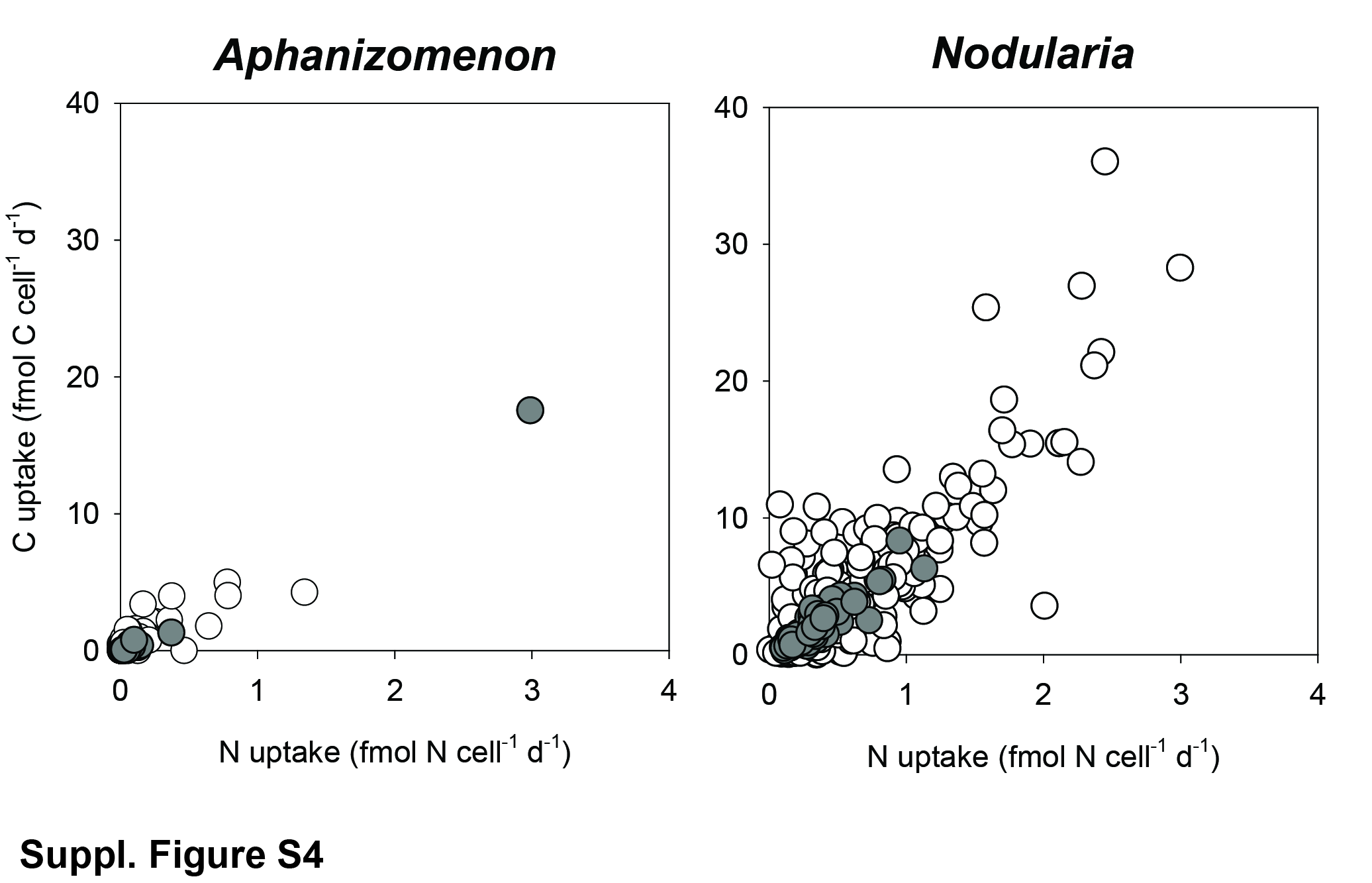

Supplement: Supplementary file 5 — Supplementary Figure S4 [file 41396_2019_453_MOESM5_ESM.tif]
